# Disease burden and quality of life in children with sickle cell disease in Italy: time to be considered a priority

**DOI:** 10.1186/s13052-021-01109-1

**Published:** 2021-07-29

**Authors:** Raffaella Colombatti, Maddalena Casale, Giovanna Russo

**Affiliations:** 1 Clinic of Pediatric Hematology Oncology, Department of Woman’s and Child’s Health, University of Padova, Padova, Italy; 2 Department of Women, Child and General and Specialized Surgery, University “Luigi Vanvitelli”, Naples, Italy; 3 Pediatric Hemato-Oncology Unit, Department of Clinical and Experimental Medicine, University of Catania, Catania, Italy

**Keywords:** Sickle cell disease, Child, Adolescent, Disease burden, Quality of life, Italy

## Abstract

The objective of the present article is to highlight the need for attention to Quality of Life of patients with Sickle Cell Disease living in Italy. The transformation of sickle cell disease from a severe life-threatening disease of childhood into a chronic, lifelong condition due to the significant improvements in care and treatment options, imposes increasing new challenges to health care providers and patients. Patients now face physical, psychosocial and emotional challenges throughout their lives. They generally have to receive chronic treatments and regular multidisciplinary monitoring which increase social and emotional burden rendering adherence to treatment sometimes complicated. A chronic disease impacts all aspects of patients’ lives, not only the physical one, but also the social and emotional aspects as well as the educational and working life. The entire “Quality of Life” is affected and recent evidence demonstrates the importance quality of life has for patients with chronic illness. The results of this review focus on emerging data regarding quality of life across the lifespan of patients with Sickle Cell Disease, and highlight the need for more action in this field in Italy, where recent immigration and improved care determine an increasing population of children with sickle cell disease being taken into long term care.

## Introduction

Sickle cell disease (SCD) is the most common genetic disorder worldwide. Every year, an average of 300,000 children are born with sickle syndromes, and the prevalence of sickle cell among newborns ranges from 0.1/1000 in non-endemic countries to 20/1000 in several parts of Africa [1]. At the basis of SCD pathophysiology are vaso-occlusion, chronic hemolytic anemia and vasculopathy that cause both acute and chronic complications, which have a profound impact on the quality of life (QoL). In spite of the improved survival of children with SCD in the past decades due to significant advances in clinical care in developed countries, painful vaso-occlusive crises, recurrent admissions to health care facilities and long hospital stays disrupt the social and school life of children and adolescents with SCD causing a poor QoL, compared to their peers [2, 3].

Generic and disease-specific measures of QoL have been developed and validated in chronic illnesses to measure the influence of health on functional domains, such as physical QoL, mental QoL, fatigue, pain, social engagement, relationships, and emotional distress. Examples such as the Child Health Questionnaire, Pediatric Quality of Life Sickle Cell Disease Module (PedsQL SCD), Patient-Reported Outcomes Measurement Information System (PROMIS) have been used in SCD and have documented various levels of impaired QoL, mainly in the United States [4, 5]. Adolescents report poor sleep quality, moderate levels of fatigue and that sleep quality mediates the relationship between pain and fatigue [6]. Children with SCD report also substantial problems with physical functioning, pain and sleep during and immediately following vaso-occlusive crises [7]. Depression among adolescents is common [8] and socio-economic factors as well as racism play an additional role in reducing QoL [2, 9, 10].

## Italian experience with SCD

Historically SCD in Italy was a disease limited to the population of Sicily where the sickle cell allele frequency is between 2 and 13% [11]. Immigration from Africa, South America, and the Balkans since the early 2000s, mainly into the northern regions of the country, has changed the geographic profile of SCD, with an increase of new diagnoses in the northern part of the country, making this disease a widespread public health issue [12, 13]. As a consequence of the migratory movements, in the past 15 years the number of children with SCD has progressively increased in Italy, posing many challenges to pediatricians and hematologists [14]. Although the fragmentation of the Italian Health System in 20 Regional Health Systems and the lack of a National SCD Newborn Screening Program as well as the lack of a widespread National SCD Registry do not allow to give accurate data on the number of adults and children with SCD currently living in Italy, estimates can be derived from published data coming from Regional Registries, Local Pilot Newborn or Antenatal Screening Programs or from Surveys or Clinical Studies published by centers belonging to national scientific societies like the Italian Association of Pediatric Hematology Oncology (AIEOP) and the Italian Society of Thalassemia and Haemoglobinopathies (SITE). Some of the publications refer to nationwide studies in children, adults or both [13–18] and allow to estimate around 1000–1500 children and 2500–3000 patients overall with SCD living in Italy.

Admissions of children with SCD for acute events like vaso-occlusive crisis and acute chest syndrome are common in Italy [13, 17–20] and cognitive problems have also been described [21, 22]. A national network was developed and clinical recommendations tailored to the Italian setting were produced by the Italian Association of Pediatric Hematology Oncology (AIEOP) and an effort to ensure children and adolescents with SCD living in Italy received disease modifying treatment like hydroxyurea was made [18].

Currently, and differently from the adult population with SCD who are mainly native Italians, children with SCD living in Italy are mainly first- or second-generation immigrants coming from English and French Speaking African Countries, therefore belong to socially vulnerable groups. Linguistic, racial and socio-economic factors worsen QoL in immigrant children with chronic illness in Italy, in spite of the free health care system in the country. Hence the need to specifically explore QoL for children with SCD in our setting.

## Existing literature on QoL and SCD in Italy

The PubMed, Cochrane Library, Springer databases were searched for articles published up to January 26th 2021 including in various combinations the terms: quality of life, disease burden, child, children, sickle cell disease, sickle cell anemia, Italy. No language restriction was given. Additional websites of patients’ organizations and Google were searched for articles in Italian.

Having not found specifically articles describing the Italian situation, a general search on quality of life and disease burden in children with sickle cell disease was performed, beyond the articles considered to address the issue in the Introduction. The authors also searched the references from relevant identified articles. A review of the national guidelines currently implemented in Italy was also performed to check if a specific mention is made regarding QoL monitoring.

There were no manuscript describing specifically disease burden and QoL for children or adolescents with SCD living in Italy. However, a reduction in the number of painful crisis and in the number of admissions, which are generally linked to QoL, was reported for children on Hydroxyurea therapy [18]. Moreover, there were also no data regarding QoL of parents or caregivers of children with SCD living in Italy. Nevertheless, a single center study investigating parental stress in relationship to chronic hematological disorders, included also parents of children with SCD and reported higher stress among parents of children with SCD compared to those of children with Immune Trombocytopenic Purpura [23]. Italian patients and caregivers participated also in the International Sickle Cell World Assessment Survey (SWAY) in which emotional and wellbeing were accessed globally through a dedicated survey: significant impact of SCD on clinical, emotional, psychological and social aspects of life were reported [24].

No mention is given to QoL in the current National Recommendations for the Management of Children With Sickle Cell Disease in Italy [25] and this should probably be amended.

## Comment

The results demonstrate the lack of data regarding quality of life of children, adolescents with SCD and their caregivers in Italy. Interestingly, the lack of data concerns also adults with SCD, with no information also available regarding QoL specifically for adults with SCD in Italy.

This finding is not surprising, since SCD is a recent disease in Italy, with National Recommendations and dedicated networks of pediatric care being organized only since the early 2000s [14, 25]. It is reasonable that pediatricians and pediatric hematologists sought to address first the more urgent health related issues, like the organization of SCD specialized comprehensive care and detailed clinical pathways for acute and chronic conditions [14, 25].

Language barriers and cross-cultural limitations could also have played a role in limiting the research or the interest in addressing QoL overall in patients with SCD in Italy since SCD is a disease involving primarily immigrant children with SCD and their families and interactions beyond the necessary health care are generally more complex. Nevertheless, the lack of specific research in QoL concerns also children with Thalassemia, with very limited amount of data available from the literature and only on disease burden and QoL of adults with Thalassemia [26], in spite of Italy being one of the countries in the world with the widest population of patients with this disease. These lack of data on disease burden and QoL in SCD probably underscores the need for a call to general awareness among pediatricians and hematologist of the importance of QoL and disease burden evaluation as necessary outcomes to be considered in the adequate care of SCD in Italy, as it already happens in other countries. It is worth mentioning that limited evidence of QoL for patients with SCD is available from other European Countries and only for adults [27], highlighting the need for additional research in health related QoL in SCD to continue to advance our knowledge in this area, including more-direct and contemporary comparisons with other diseases.

The voice of the SCD patients living in Italy also points towards this direction. In fact, QoL was identified as the second item among the Top Ten Research Priorities developed within the framework of the Patient Advocacy Group project at the Annual Academy of Sickle cell Disease and Thalassemia Conference in London 2019 (2nd Top: How can we improve quality of life for people with SCD?) to which also Italian Representatives contributed [28] and was underscored also at the second Global Patient Advocacy Group Virtual Meeting in October 2020, again with the participation of Italian Representatives (https://eurobloodnet.eu/news/168/sickle-cell-disease-patients-and-parents-patients-educational-session-at-ascat-2020-a-joint-project-of-ern-eurobloodnet-ascat-and-bsh). Moreover, Italian teenagers and adults with SCD as well as guardians were among the global population of patients completing the International Sickle Cell World Assessment Survey (SWAY) [24] in which the improvement of QoL was ranked as the first top treatment goal in the 6–11, 12–16 and 17–18 year old age groups. Adolescents with SCD living in Italy have also demonstrated a high completion rate of the daily electronic patient-reported outcome (PRO) diary in the international DOVE trial [29] further underscoring their interest in being involved to report PROs.

All the validated and standardized QoL questionnaires are also available in the Italian language and pediatric hematologists have recently been involved in QoL evaluation in children with non-malignant disorders such as Hemophilia [30, 31] and Chronic Immune Thrombocytopenia [32], with significant participation from patients/caregivers and important information provided.

## Conclusion

The improvement of health related QoL is a major objective in health care and research on any chronic disease, including SCD. The time has come to consider standard QoL and disease burden evaluation as part of routine comprehensive care for children and adolescents with SCD living in Italy. The fact that the majority of children and adolescents with SCD are first- or second-generation immigrants coming from African English or French speaking countries, although a necessary factor to be taken into consideration when performing standardized questionnaires assessment, should not limit the evaluation of QoL or disease burden. Recent data coming from several African countries shows the feasibility and success of taking QoL evaluation into account [33, 34].

The appearance of new treatment options for SCD, including not only new drugs as disease modifying treatments, but also gene therapy among the disease curative treatments, highlights the need to increase the effort to improve QoL evaluation in the clinical and research setting. The time has come to do so in Italy too. The characteristics of the pediatric population with SCD living in Italy, mainly immigrants from English and French African countries, highlights the need to identify adequate instruments for QoL evaluation, including PROs and to consider linguistic, social and economic factors into account when interpreting the results.

## Data Availability

Not applicable.

## Abbreviations

PedsQl: Pediatric Quality of Life Sickle Cell Disease module
PROMIS: Patient-Reported Outcomes Measurement Information System
PRO: Patient Reported Outcome
QoL: Quality of life
SCD: Sickle cell disease
SWAY: Sickle Cell World Assessment Survey
